# A 15-Year Single-Institution Retrospective Study of Primary Pancreatic Cancer Treated with Non-Ablative Palliative Radiotherapy

**DOI:** 10.3390/cancers16050881

**Published:** 2024-02-22

**Authors:** Randa Kamel, Tinghua Zhang, Suzanne Comino, Kristopher Dennis

**Affiliations:** 1 Department of Radiation Oncology, UZ Brussel, Vrije Universiteit Brussel, Jette, 1090 Brussels, Belgium; 2 Ottawa Hospital Research Institute, Ottawa, ON K1H 8L6, Canada; 3 Radiation Medicine Program, The Ottawa Hospital, Ottawa, ON K1H 8L6, Canada; scomino@toh.ca; 4 Division of Radiation Oncology, The Ottawa Hospital, The University of Ottawa, Ottawa, ON K1H 8L6, Canada

**Keywords:** locally advanced pancreatic cancer, primary pancreatic cancer, palliative radiotherapy, single-institution retrospective cohort study, palliative pancreatic cancer radiation

## Abstract

**Simple Summary:**

Most pancreatic cancer patients present with locally advanced tumors non-amenable to surgical resection or other radical treatments. The most used form of radiotherapy in those cases is palliative-intended non-ablative radiotherapy, mainly to alleviate the significant symptoms caused by the local progression of pancreatic primaries. Research in this area is severely under-represented in the literature and the guidelines for palliative radiotherapy dose prescriptions in this setting are based on very scarce data. The paucity of studies in this area contrasts with that for palliative radiotherapy for bone, brain, liver, and lung diseases, where various radiotherapy strategies have been evaluated, and evidence-based recommendations guide practice. An early step toward optimizing palliative radiotherapy use for pancreatic cancer is quantifying and describing its current use. Here, we describe the use of non-ablative palliative radiotherapy among patients treated for primary pancreatic tumors in our institution over 15 years.

**Abstract:**

We studied the use of palliative radiotherapy (RT) among patients with primary, non-curable, locally advanced pancreatic cancer. In this subset of patients, with very poor survival, various palliative RT dose fractionation schemes are used; but, in the absence of a guideline, practice patterns vary, and dose choice is mainly based on the physician’s intuition. We divided the patients into three groups, according to the dose fractionation schedules received: low (A), intermediate (B), and high (C) dose groups, to study the potential differences in outcome between the different dose prescriptions. Cohort: *n* = 184. Median age: 69 years. Male: *n* = 105 (57%), female: *n* = 79 (43%). Stage IV: *n* = 117 (64%). T4: *n* = 127 (69%). Tumor location: head: *n* = 109 (59%), body: *n* = 37 (20%), tail: *n* = 25 (14%), neck: *n* = 11 (6%), and uncinate: *n* = 2 (1%). Prior systemic therapy: *n* = 66 (36%). Most common dose fractionations received: 20 Gy in five fractions *n* = 67 (36%), 30 Gy in 10 fractions *n* = 49 (27%), and 8 Gy in one fraction *n* = 23 (13%). Group A: *n* = 33 (18%), median overall survival (OS) 19 days (95% CI 4–33). Group B: *n* = 84 (46%), median OS 52 days (95% CI 43–60). Group C: *n* = 67 (36%), median OS 126 days (95% CI 77–174). Median days to in-field progression: Group A 59 days (range 7–109), Group B 96 days (range 19–173), and Group C 97 days (range 13–475). To our knowledge, this is the largest reported retrospective cohort of patients receiving non-ablative palliative RT to treat their primary pancreatic tumors. Most patients had metastatic disease, T4 tumors of the pancreatic head and had not received prior systemic therapy. A significant survival benefit was seen favoring the high dose/longer RT fractionation group, presumably due to appropriate patient selection rather than an RT effect. Despite the relatively short median overall survival, one fifth of the patients were found to experience an in-field progression following RT.

## 1. Introduction

Most patients with pancreatic cancer have poor oncologic outcomes [1,2]. Some receive no anti-cancer therapy, and a minority are candidates for curative-intent treatment protocols involving surgery. Chemotherapy is the mainstay treatment for most patients [3,4]. The role of non-ablative radiotherapy (RT) in neoadjuvant, adjuvant, and radical settings is still evolving, and the role of ablative RT in any setting is still uncertain [5]. Owing to these uncertainties surrounding RT practice, as well as the aggressive nature of the disease, the often poor functional status of affected patients, and the anatomical, logistical, and resource-related factors, all act as barriers to getting access to ablative RT. So, the most common form of RT administered to primary pancreatic tumors in many jurisdictions is conventionally fractionated or hypo-fractionated non-ablative palliative-intent RT.

Unfortunately, research involving patients treated in this manner is severely under-represented in the literature, despite the significant symptoms caused by the local progression of pancreatic primaries [6]. The 2021 guidelines on pancreatic adenocarcinoma from the National Comprehensive Cancer Network (NCCN) state that palliative RT, typically 25–36 Gy in 2.4–5 Gy fractions, with or without concurrent chemotherapy, can be used to help alleviate pain, bleeding, and obstruction [7]. It references a single 26-patient study that incorporated concurrent chemotherapy with hypo-fractionated RT [8]. The 2019 guidelines on pancreatic cancer from the American Society for Radiation Oncology (ASTRO) strongly recommend palliative RT to the primary site for symptomatic patients and suggests regimens of 20 Gy in five fractions or 30 Gy in 10 fractions [5]. It references four studies that cumulatively reported on less than 60 patients treated with non-ablative RT alone [9,10,11,12]. Both the NCCN and ASTRO guidelines acknowledge the limited data supporting their recommendations and advise practitioners to take into account a patient’s metastatic burden, performance status, and estimated survival, when choosing a dose fractionation.

The potential benefits of palliative RT for these patients include reducing pain and bleeding, providing local control, and relieving gastric outlet or biliary obstruction. Conversely, well-known acute toxicities following RT to the upper abdomen include nausea, retching, emesis, cramping, diarrhea, gastritis, and fatigue. But with limited data to guide practice, one must weigh the benefits of RT against the known toxicities and inconveniences [13,14,15]. The paucity of studies in this area contrasts with that for palliative RT for bone, brain, liver, and lung disease, where various RT strategies have been evaluated in these settings, and evidence-based recommendations guide practice.

Optimal doses and durations of palliative RT for pancreatic cancer have yet to be determined. To date, practice patterns vary according to the physician’s intuitive choice. These knowledge gaps are problematic because the goals of treatment should be to relieve the patient’s symptoms and maintain or improve their quality of life, all while inconveniencing them as little as possible.

An important early step toward optimizing palliative RT for patients with pancreatic cancer is the quantification and description of the outcomes from its current use. Herein, we report the experience from our institution over 15 years. We aimed to describe how these tumors responded to the different dose fractionation schedules prescribed. We initially hypothesized, due to the known limited life expectancy in this subset of patients, that single or brief fractionation schedules might deliver similar outcomes to longer courses and, hence, be more cost effective and more convenient for the patients.

## 2. Materials and Methods

This single-institution retrospective study was carried out in a tertiary-level Canadian center and the exclusive provider of radiotherapy services to a population of 1.4 million residents. The Research Ethics Board approved the study protocol on the 8 April 2020. Our institutional electronic records were searched for patients that began courses of non-ablative palliative RT during the period from 1 January 2005 until 31 December 2019. The RT records from prior to this period were not stored electronically. Patients were eligible for analysis if the RT was directed at their unresected primary pancreatic cancer with a total dose ≤ 40 Gy. Patients were ineligible if they received concurrent systemic therapy or were undergoing treatments for post-surgical locoregional recurrences. The last date of potential follow up was 1 February 2021.

As previously mentioned, different palliative RT dose fractionation schedules exist and are used for treatments in this setting. In this cohort, we have seen these variations used in prescriptions by our treating physicians. We believe that they intuitively made their treatment schedule choice due to meaningful differences in the patients’ phenotypes, fitness levels, and overall palliative goals.

**Figure 1:**
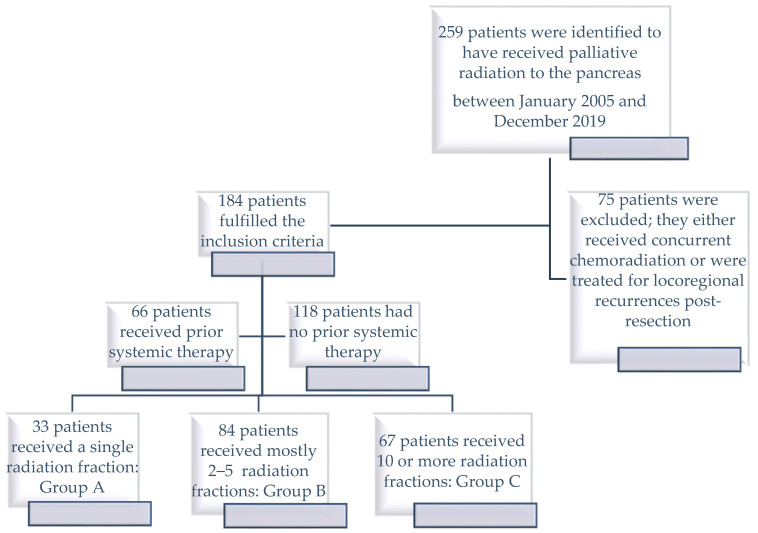
A diagram showing the number of included and excluded patients and the treatments received.

We wanted to check whether single fractionation regimens or brief fractionation schedules might deliver similar outcomes to the higher doses and long fractionation courses. So, patients were grouped according to the dose fractionation schedules they received: Group A contained patients who received a single fraction, Group C contained patients who received ≥30 Gy in 10 or more radiation fractions, and Group B contained patients who received mostly between 2 and 5 radiation fractions combining all dose fractionation intensities between those from Groups A and C (Figure 1).

Our primary points of interest included overall survival and local control. Other outcomes of interest included demographic, clinical, systemic therapy details, RT details, and any reported toxicities, or quality of life changes.

Performance status at the time of initial radiation oncology consultation was abstracted when available, and retrospectively assigned when not available. Baseline diagnostic images and reports were reviewed to assign the TNM values, according to the AJCC 8th edition system for pancreatic ductal adenocarcinomas. Descriptive statistics were used to summarize the data, including the medians and ranges for continuous variables and the frequencies and proportions for categorical variables.

The Kaplan–Meier method was used for time-to-event analyses using the SPSS statistical software, IBM SPSS Statistics, Version 28.0. Armonk, NY, USA. The survival rate was calculated from the start of the patient’s first RT course until death from any cause, and the patients were censored at the last follow up if still alive. The in-field progression-free survival was calculated from the end of the patient’s first RT course until radiographic confirmation of an in-field progression, and the patients were censored at the last follow up if there was no evidence of an in-field progression. For the patients that began RT without evidence of distant metastases, the distant progression-free survival was calculated from the end of the patient’s first RT course until radiographic confirmation of distant progression, and the patients were censored at the last follow up if there was no evidence of a distant event. The *p*-value was considered statistically significant if ≤0.05.

## 3. Results

One hundred and eighty-four patients satisfied the inclusion criteria and formed the study cohort (Table 1). The median age at the time of RT commencement was 69 years (range 33–92). More patients were male (*n* = 105/184 (57%)) than female (*n* = 79/184 (43%)). The ECOG performance status was abstracted for 83/184 patients (45%) and retrospectively assigned for 101/184 patients (55%). Most patients were ECOG 2 (*n* = 63/184 (34%)) or ECOG 3 (*n* = 74/184 (40%)). At RT commencement 44/184 (24%) of patients were admitted to hospital. Of the 144 patients that began RT as outpatients, only four patients (3%) required hospitalization prior to the completion of their RT treatment course (two of these patients belonged to Group B and the other two belonged to Group C).

Most patients had T4 primary tumors (*n* = 127/184 (69%)), and most primary tumors were located in the pancreatic head (*n* = 109/184 (59%)) or body (*n* = 37/184 (20%)). Almost half of the patients were node positive (N+) at diagnosis (*n* = 86/184 (47%)). And most had distant metastases (M1) at diagnosis (*n* = 117/184 (64%)). Common bile duct stents were present in (*n* = 90/184 (49%)) patients and duodenal stents were present in (*n* = 3/184 (2%)) patients. Systemic therapy had been received at some point prior to RT commencement by just over a third of patients (*n* = 66/184 (36%)). The median time from the last day of receiving systemic treatment until the date of RT commencement was 44 days (range 6–419 days). The most used chemotherapy agents prior to RT were gemcitabine, FOLFIRINOX, and Abraxane.

The fractionation schedules ranged in intensity from 8 Gy in a single fraction to 40 Gy in 15 fractions (Table 2). The most common fractionations received were 20 Gy in five fractions (*n* = 67/184 (36%)), 30 Gy in 10 fractions (*n* = 49/184 (27%)), and 8 Gy in one fraction (*n* = 23/184 (13%)). Of the 155 patients that were due to receive a multiple fraction treatment regimen, 15 (10%) patients were not able to continue the RT course due to a deterioration in their general condition and their treatment had to be prematurely terminated. The patients were assigned to groups as follows: Group A (*n* = 33/184 (18%)), Group B (*n* = 84/184 (46%)), and Group C (*n* = 67/184 (36%)) (Table 2). Most treatment planning CT scans were free breathing (*n* = 171/184 (93%)) rather than 4D (*n* = 13/184 (7%)). Most patients were treated with 3D conformal RT (*n* = 95/184 (52%)) or IMRT/VMAT (*n* = 62/184 (34%)). Of the 164/184 (89%) plans created with target volumes, a GTV was contoured in 91/164 (55%) treatment plans, a CTV in 68/164 (41%), a VOI in 28/164 (17%), an ITV in 4/164 (2%), and a PTV in 149/164 (91%). The use of IMRT increased over the 15-year period of the analysis, applied in one third of our cohort; VMAT was essentially the form of IMRT used. Only one patient received a second course of RT; only information from the first course of treatment was used for analysis.

The documentation on the specific goals of RT, the symptoms, and the supportive care interventions were inconsistent and seen in only 117/184 (64%) of patients’ charts. Most patients were receiving opioids at the time of their initial consultation (*n* = 151/184 (82%)). As the RT progressed, the symptoms (whether tumor or RT-induced) were documented for 15/33 (45%) of the patients from Group A, 54/84 (65%) of the patients from Group B, and 48/67 (72%) of the patients from Group C. The most commonly mentioned symptoms were fatigue and nausea. At least one post-RT follow-up appointment with a radiation oncologist occurred for 85/184 (46%) patients, with the median number of follow-up appointments being one (range 1–5).

**Figure 2:**
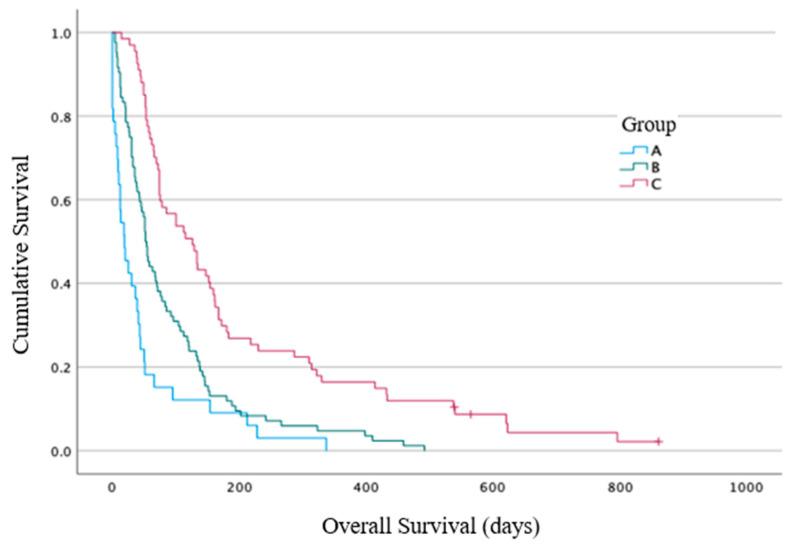
Overall survival from the time of radiotherapy commencement, calculated in days.

**Figure 3:**
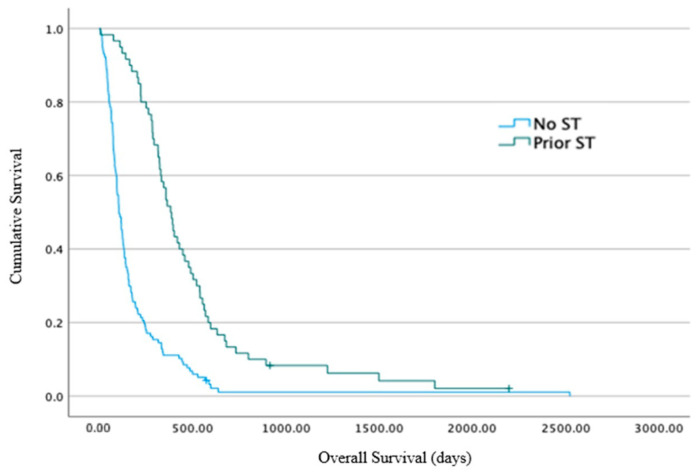
Post-hoc overall survival, calculated in days, from the time of pathological diagnosis for patients who received or did not receive systemic therapy prior to radiotherapy. ST = systemic therapy.

The primary end point results: The median overall survival (OS) from the time of RT commencement for Group A was 19 days (range 0–377 days, 95% CI 4–33 days), for Group B it was 52 days (4–492 days, 95% CI 43–60 days), and for Group C it was 126 days (14–861 days, 95% CI 77–174 days) (Figure 2). A statistically significant difference between the three groups for the OS was identified using log-rank analysis (*p* < 0.001). The hazard ratio (HR) for death for Group B vs. Group A was 0.5 (95% CI 0.3–0.8), *p* = 0.003, and for Group C vs. Group A was 0.26 (95% CI 0.2–0.4), *p* < 0.001. The median OS for the entire cohort (*n* = 184) was 62 days (range 0–861 days, 95% CI 49–74 days). Post-hoc exploratory analysis showed that the median OS from the time of pathological diagnosis was greater for patients that had received systemic therapy prior to RT (*n* = 66/184 (36%)) compared to those that had not (*n* = 118/184 (64%)): the median survival was 381 days (74–2196 days, 95% CI 332–429 days) vs. 104 days (range 4–2523 days, 95% CI 84–123 days), *p* < 0.001 (Figure 3).

**Figure 4:**
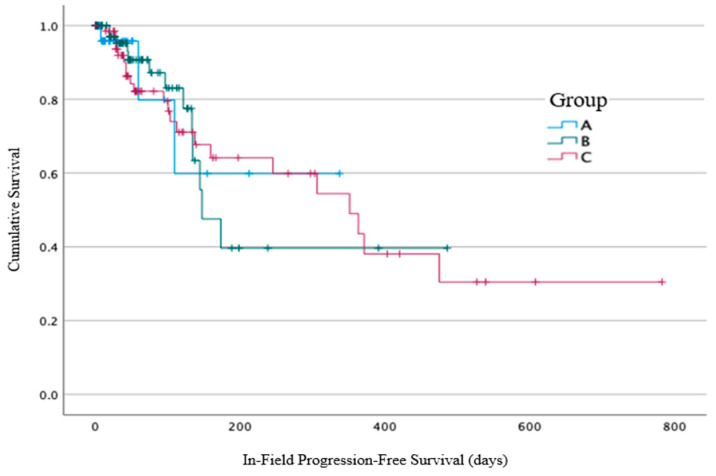
In-field progression-free survival from the end of radiotherapy, calculated in days from RT end.

In-field local progression was identified in 38/184 (21%) of patients: 3/33 (9%) of patients from Group A, with a median time from the end of the RT to the progression of 59 days (7–109 days), compared to 13/84 (15%) of patients from Group B, with a median time of 96 days (range 19–173 days), and 22/67 (33%) of patients from Group C, with a median time of 97 days (range 13–475 days) (Figure 4). No statistically significant difference between the three groups for in-field local progression was identified using log-rank analysis (*p* = 0.99).

**Figure 5:**
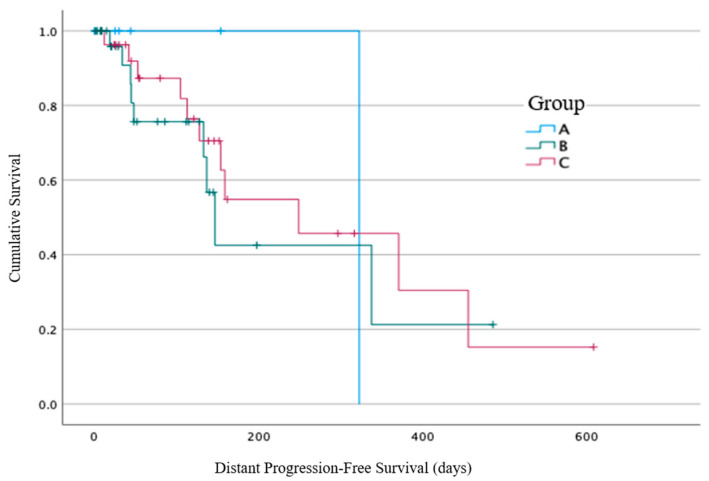
Distant progression-free survival from the end of radiotherapy, calculated in days from RT end.

At the time of the RT commencement, 65/184 (35%) patients had no confirmed evidence of metastatic disease: 6/33 (18%) from Group A, 32/84 (38%) from Group B, and 27/67 (40%) from Group C (Figure 5). Within these subgroups, 1/6 (17%) patients from Group A had distant progression identified at 337 days from the end of RT (the other five patients died within 30 days), compared with 9/32 (28%) patients from Group B with a median time of 147 days (19–338 days, 95% CI 124–169 days), and 11/27 (41%) patients from Group C with a median time of 249 days (42–456 days, 95% CI 81–126 days). No statistically significant difference between the three groups for distant progression was identified using log-rank analysis (*p* = 0.73).

## 4. Discussion

To our knowledge, this is the largest reported cohort of patients with pancreatic cancer that received non-ablative palliative RT on their primary tumors. Our results improve our understanding of the physicians’ practice patterns in this setting and the natural history of patients treated in this manner. Our cohort comprised mainly of patients with poor performance status, with T4 disease involving the head of the pancreas, where local symptoms and complications, such as obstruction, bleeding, and pain, can first necessitate local therapies, such as stenting and RT, regardless of the disease burden, regardless of the metastatic status, and regardless of the ultimate plans for systemic therapy administration. The majority of our patient referrals for palliative RT have been received from the medical oncologists, the presumed ‘gate keepers’ for patients with advanced pancreatic cancers.

We found that patients who received longer RT courses lived longer than those that received shorter courses. This difference probably reflects proper patient selection for longer fractionation schedules, rather than an effect of the RT delivered. As with other cohorts of patients with aggressive and advanced cancers, prognostication is critical when prescribing palliative RT for patients with pancreatic cancer. Future studies that examine extended cohorts of patients will hopefully create tools to help with this important exercise and will eventually help build a proper patient categorization model. Comparing patients as a function of the duration of the RT courses they received, rather than according to the biologically effective dose (BED), and the separation of the groups’ survival curves seems to suggest that this was a reasonable method for identifying subgroups of patients with clinically meaningful differences, including survival expectations.

Our observed median OS of 62 days was similar to figures reported in other retrospective series. Wolny-Rokicka and colleagues reported a 1-year OS rate of only 16% among 31 patients treated with 6–30 Gy over 1–10 fractions [16], while Ebrahimi and colleagues reported a median survival of 3.5 months among 61 patients treated with 1–3 weekly fractions of 8 Gy [17].

Unfortunately, the retrospective nature of our data limits our ability to accurately analyze the toxicity outcomes, because their ratings are retrospectively assigned. The specific palliative goals of the RT were not well described in most clinical notes, e.g., pain relief, and/or hemostasis, and/or short-to-long term local control. And owing to the short life expectancy, only a minority of patients had planned follow-up appointments. Understanding the likelihood of and latency to symptom control following RT and documenting iatrogenic toxicities should be a priority for future works, so that we can better counsel patients on the potential benefits and toxicities of treatments. Simple prospective observational studies could follow patients to collect these important outcome measures, with precise pre- and post-RT pain scoring, along with quality-of-life data from validated questionnaires. Morganti and colleagues performed such a highly controlled cohort study among patients that received 30 Gy in 10 fractions [18]. They documented a median overall survival of 7.5 months and a 75% pain response rate at 4 weeks following RT, but the study was small, consisting of only 12 patients. Correlating patient-reported outcomes with organ-at-risk dosimetric data could also be a useful exercise; a relative knowledge gap in our understanding of the patient experience is the consequence of lower ‘splash’ doses to abdominal structures, such as the small and large bowel and the bowel bag. Improving our understanding of the impact of the lower end of the DVH curve could benefit future patients that receive both non-ablative and ablative RT.

The use of IMRT and VMAT increased over time, but only a third of our cohort received treatments delivered with these techniques. Documenting the potential benefits of highly conformal techniques for palliative RT should also be prioritized in future studies, as their use in the curative setting has been shown to be associated with reductions in the doses administered to adjacent organs at risk and improvements in upper gastrointestinal toxicity outcomes compared to simpler techniques [19,20,21,22,23].

Despite the short survival rates in this cohort, an in-field progression was identified in a fifth of patients. This is not surprising following single fraction RT, as doses in this range have the potential to provide hemostasis and pain relief but are not likely to result in local control. However, treatment failures were seen among patients that lived longer and had received multiple fraction regimens as well. Interestingly the median time to in-field progression was essentially the same for Groups B and C. It may be that there is not a dose response for local control within the range of fractionations studied here. And that the almost identical time to local progression could probably be due to the overlapping values of the biologically effective doses used in those two groups. Stereotactic radiotherapy given in short duration regimens may have a useful role to play in ensuring local control among patients with locally advanced pancreatic cancer with a more favorable prognoses, or even in elderly patients and those with a more tenuous performance status, so long as the treatment goals and acceptable risks are aligned between the patient and their care teams [24,25,26,27,28,29,30].

Limitations of the retrospective data warrant comment. The in-field and distant progression rates should be considered hypothesis generating rather than truth, as no protocoled post-RT follow up or imaging schedules were followed. We believe more events occurred than were documented. The performance status was also assigned retrospectively for almost half of our cohort. The symptom control outcomes were not reliably and consistently documented, limiting the ability of our patients to serve as a historical base for comparisons with future prospective cohorts for these specific outcome measures.

## 5. Conclusions

To our knowledge, this study represents the largest reported retrospective cohort of patients with pancreatic cancer that received non-ablative palliative RT to their primary tumors. Most patients had metastatic disease, had not received prior systemic therapy, and had T4 tumors of the pancreatic head. Patients treated with longer RT fractionation schedules lived longer than those treated with shorter schedules, presumably due to appropriate patient selection rather than an effect of RT. Despite the relatively short median overall survival, a fifth of the patients were found to experience an in-field progression following RT.

## Figures and Tables

**Table 1:** Characteristics of the study cohort (*n* = 184).

Characteristic		*n*	(% of 184 Patients)
**Age**	Median 69 years, range 33–92		
**Gender**	Male	105	(57)
	Female	79	(43)
**Performance Status**	ECOG 0	2	(1)
	ECOG 1	42	(23)
	ECOG 2	63	(34)
	ECOG 3	74	(40)
	ECOG 4	3	(2)
**Hospital Status**	Inpatient	44	(24)
	Outpatient	140	(76)
**TNM**	T2	6	(3)
	T3	28	(15)
	T3/4	21	(11)
	T4	127	(69)
	N0	77	(42)
	N1	72	(39)
	N2	14	(8)
	Nx	21	(11)
	M0	65	(35)
	M1	117	(64)
	Mx	2	(1)
**Tumor Location**	Head	109	(59)
	Neck	11	(6)
	Uncinate	2	(1)
	Body	37	(20)
	Tail	25	(14)
**Histology**	Adenocarcinoma	176	(96)
	Neuroendocrine carcinoma	1	(<1)
	Non-small cell carcinoma	1	(<1)
	Squamous cell carcinoma	1	(<1)
	Uncertain	5	(3)
**Stents In Situ**	Common bile duct	90	(49)
	Duodenum	3	(2)
**Prior Systemic Therapy**	Yes	66	(36)
	No	118	(64)
**Referring Physician**	Medical oncologist	86	(47)
	Surgical oncologist	49	(27)
	General practitioner	36	(20)
	Other	13	(7)

**Table 2:** Patient groupings and radiotherapy data.

	Total Dose Received	Fractions Received	*n*	(% of 184 Patients)
**Group A (*n* = 33)**	4 Gy	1	1	(0.5)
	6 Gy	1	1	(0.5)
	8 Gy	1	23	(13)
	8.5 Gy	1	2	(1)
	10 Gy	1	6	(3)
**Group B (*n* = 84)**	6 Gy	2	1	(0.5)
	8 Gy	2	3	(2)
	16 Gy	2	2	(1)
	9 Gy	3	1	(0.5)
	18 Gy	4	1	(0.5)
	20 Gy	5	67	(36)
	25 Gy	5	4	(2)
	21 Gy	7	3	(2)
	24 Gy	8	1	(0.5)
	25 Gy	10	1	(0.5)
**Group C (*n* = 67)**	30 Gy	10	49	(27)
	35 Gy	10	5	(3)
	36 Gy	15	2	(1)
	40 Gy	10	1	(0.5)
	40 Gy	15	10	(5)

## Data Availability

Data are available upon reasonable request to the corresponding author.
